# Palmitoylethanolamide Modulates GPR55 Receptor Signaling in the Ventral Hippocampus to Regulate Mesolimbic Dopamine Activity, Social Interaction, and Memory Processing

**DOI:** 10.1089/can.2016.0030

**Published:** 2017-01-01

**Authors:** Cecilia Kramar, Michael Loureiro, Justine Renard, Steven R. Laviolette

**Affiliations:** ^1^ Department of Anatomy and Cell Biology, The Schulich School of Medicine & Dentistry, University of Western Ontario, London, Ontario, Canada.; ^2^ Department of Psychiatry, The Schulich School of Medicine & Dentistry, University of Western Ontario, London, Ontario, Canada.; ^3^ Department of Psychology, The Schulich School of Medicine & Dentistry, University of Western Ontario, London, Ontario, Canada.

**Keywords:** PEA, GPR55, cannabinoid, dopamine, social interaction, ventral hippocampus

## Abstract

**Introduction: ** The GPR55 receptor has been identified as an atypical cannabinoid receptor and is implicated in various physiological processes. However, its functional role in the central nervous system is not currently understood. The presence of GPR55 receptor in neural regions such as the ventral hippocampus (vHipp), which is critical for cognition, recognition memory, and affective processing, led us to hypothesize that intra-vHipp GPR55 transmission may modulate mesolimbic activity states and related behavioral phenomena. The vHipp is involved in contextual memory and affective regulation through functional interactions with the mesolimbic dopamine system.

**Materials and Methods:** Using a combination of *in vivo* electrophysiology and behavioral pharmacological assays in rats, we tested whether intra-vHipp activation of GPR55 receptor transmission with the fatty acid amide, palmitoylethanolamide (PEA), a lipid neuromodulator with agonist actions at the GPR55 receptor, may modulate mesolimbic dopaminergic activity states. We further examined the potential effects of intra-vHipp PEA in affective, cognitive and contextual memory tasks.

**Discussion:** We report that intra-vHipp PEA produces a hyper-dopaminergic state in the mesolimbic system characterized by increased firing and bursting activity of ventral tegmental area dopaminergic neuron populations. Furthermore, while PEA-induced activation of GPR55 transmission had no effects on opiate-related reward-related memory formation, we observed strong disruptions in social interaction and recognition memory, spatial location memory, and context-independent associative fear memory formation. Finally, the effects of intra-vHipp PEA were blocked by a selective GPR55 receptor antagonist, CID160 and were dependent upon NMDA receptor transmission, directly in the vHipp.

**Conclusions:** The present results add to a growing body of evidence demonstrating important functional roles for GPR55 signaling in cannabinoid-related neuronal and behavioral phenomena and underscore the potential for GPR55 signaling in the mediation of cannabinoid-related effects independently of the CB1/CB2 receptor systems.

## Introduction

The endocannabinoid system is implicated in numerous physiological phenomena, such as emotional homeostasis, stress responsiveness, cognition, and memory.^1–4^ The observation that cannabinoids can produce effects independently of the canonical CB1 and CB2 receptor (CB1/2R) systems suggests that other functional G-protein-coupled receptors (GPCRs) may interact with cannabinoid ligands.^5^ The GPR55 receptor, first cloned in 1999,^6^ has emerged as a new potential member of the endocannabinoid system.^7^ The GPR55 is phylogenetically distinct from traditional CB1/2Rs^8,9^ and it lacks the classical endocannabinoid binding pocket.^10^ While little is known regarding the central functional roles of the GPR55, it has been shown to be expressed in presynaptic terminals and is anatomically colocalized with both the CB1R^11^ and glutamatergic synaptic vesicles expressing VGLUT1 in the hippocampus.^12^ Functionally, GPR55 has been shown to modulate intracellular Ca2+ stores^13^ and recent evidence has demonstrated that GPR55 signaling directly in the hippocampus can potently regulate the release of glutamate through an N-methyl-D-aspartate (NMDA) receptor-dependent mechanism.^12^

Palmitoylethanolamide (PEA), an endogenous fatty acid amide, generates its neuromodulatory effects acting via several targets, including the peroxisome proliferator-activated receptor alpha (PPARα)^14^ and the GPR55 receptor.^15^ PEA may also indirectly activate CB1/2R.^16^ Since the initial characterization of PEA, it has been used clinically to treat intestinal inflammation^17^ and has been shown to possess neuroprotective, anti-neuroinflammatory, and analgesic properties.^18^ Despite this, very little is understood regarding the possible central effects of PEA, nor its possible modulation of cognitive or affective processing.

We have previously reported that cannabinoid CB1R transmission within the mammalian ventral hippocampus (vHipp) is critically involved in modulating both mesolimbic dopaminergic activity states, and the processing of both reward and aversion-related emotional memory and cognitive processing.^19,20^ Although both PEA and GPR55 signaling has been implicated in a wide range of physiological processes^18,21^ their potential effects in the vHipp-mesolimbic circuitry during affective or cognitive processing is currently unknown. Given the established functional role of intra-hippocampal GPR55 signaling in modulating hippocampal neuronal activity states and glutamatergic release, we hypothesized that intra-vHIPP PEA, through activation of GPR55 receptors, may modulate mesolimbic dopaminergic activity states along with affective and cognitive/memory processing behaviors.

Using *in vivo* electrophysiological recordings and behavioral pharmacological assays in rats, we examined the potential effects of intra-vHipp PEA infusion on dopamine (DA) and non-dopaminergic ventral tegmental area (VTA) neuronal activity states. Furthermore, using a battery of behavioral pharmacological assays, we examined the potential effects of vHipp PEA infusion in reward and aversion-related associative memory formation, social interaction and recognition behaviors, and novel object memory processing. We report that vHipp PEA infusion potently regulates VTA dopaminergic activity states via activation of GPR55, but not CB1Rs. Furthermore, intra-vHipp PEA-induced activation of GPR55 receptors strongly disrupted the processing of social interaction and recognition memory, spatial location memory, and context-independent associative fear memory formation, through a local NMDA receptor-dependent mechanism.

## Materials and Methods

### Rats and surgeries

Male Sprague-Dawley rats (300–350 g; Charles River, Quebec, Canada) were used in compliance with the Canadian Council for Animal Care and institutional guidelines. Rats were housed under controlled conditions (12 h light/dark cycle and food/water access *ad libitum*). For cannula implantation, rats were anesthetized with a mixture of ketamine (80 mg/mL) and xylazine (6 mg/mL) and placed in a stereotaxic device. Stainless steel guide cannula (22-gauge; PlasticsOne) were implanted bilaterally into the vHipp at the following coordinates: Anteroposterior (AP): −5.6 mm, Lateral (L): ±5.0 mm from bregma, and Dorsoventral (DV): −6.8 mm from the dural surface (Fig. 2B). Guide cannulas were held in place using jeweler's screws and dental acrylic cement. After completion of behavioral experiments, rats received an overdose of pentobarbital (240 mg/kg, i.p.) and were transcardially perfused with isotonic saline followed by 10% formalin. Brains were extracted and post-fixed 24 h before being placed in a 25% formalin-sucrose solution for 1 week. Brains were sliced (40 μm) using a cryostat and stained with Cresyl violet. Injector tips placements were localized with light microscopy. Rats with cannula placements found outside the anatomical boundaries of the vHipp, as defined by Paxinos and Watson (2007)^22^ were excluded from data analysis.

### Drug treatment and administration

The following drugs were used during behavioral and/or *in vivo* electrophysiological experiments: the GPR55 endogenous lipid agonist PEA (0.5 or 1 μg/0.5 μL; Tocris Bioscience), selective GPR55 antagonist CID 16020046 (CID160; 1 μg/0.5 μL; Tocris Bioscience), selective CB1R antagonist Rimonabant (RIM; 0.5 μg/0.5 μL; Tocris Bioscience), selective PPARα antagonist GW 6471 (GW; 10 or 100 ng/0.5 μL; Tocris Bioscience), and selective and noncompetitive NMDA receptor antagonist MK 801 (MK801; 1 μg/0.5 μL; Tocris Bioscience). All pharmacological compounds were dissolved in dimethyl sulfoxide (DMSO) and then diluted in phosphate-buffered saline (PBS) for a final 15% DMSO in PBS solution. Intra-vHipp microinfusions were performed immediately before the start of each behavioral experiment. A total volume of 0.5 μL per side was delivered via a 28-gauge microinfusion injector over a period of 1 min following drug infusion to ensure adequate diffusion from the tip.

### VTA neuronal activity recordings and analysis

*In vivo* single-cell extracellular recordings in the VTA were performed as described previously.^23^ Briefly, rats were anesthetized with urethane (1.4 g/kg, i.p.) and placed in a stereotaxic frame with body temperature maintained at 37°C. A scalp incision was made and a hole was drilled in the skull overlaying the vHipp and the VTA. For intra-vHipp a 10 μL Hamilton syringe was slowly lowered into the vHipp using the same stereotaxic coordinates described above. For intra-VTA recordings, glass microelectrodes (with an average impedance of 6–8 MΩ) filled with a 2% pontamine sky blue solution were lowered using a hydraulic micro-positioner (Kopf640) at the following coordinates: AP: −4.9 mm from bregma, L: ±0.7 mm, and DV: −7.0 to −8.5 from dural surface. Extracellular signals were amplified using a MultiClamp700B amplifier and recorded through a Digidata1440A acquisition system using pClamp10 software (Molecular Devices). Recordings were filtered at 1 kHz and sampled at 5 kHz. VTA DA neurons were identified according to well-established electrophysiological feature^24,25^: (1) a relatively long action potential width (>2.2 ms), (2) a slow spontaneous firing rate (2–10 Hz) that may include burst firing, and (3) a biphasic waveform consisting of a notch on the rising phase followed by a delayed after potential. In contrast, non-dopaminergic, VTA neurons were characterized based upon previously reported criteria: (1) a narrow action potential width (<1 ms), (2) a biphasic waveform (±), and (3) relatively fast firing rates (typically ∼10–20 Hz) and the absence of bursting activity.^26^ Electrophysiological analyses were performed using the Clampfit10 software package (Molecular Devices). The response patterns of isolated VTA DA neurons to microinfusion into the vHipp were determined by comparing the neuronal frequency rates between the 5 min pre- versus post-infusion recording epochs. Classification of drug infusion effects used a criterion of a +10% increase in firing frequency after infusion to be classified as an “increase” effect, and −10% decrease to be classified as a “decrease” effect. Neurons showing firing frequency parameters within these cutoff points were classified as “no change.” We also analyzed the bursting rates (number of burst events per minute) and the number of spike events within a burst. The onset of a burst was defined as the occurrence of two consecutive spikes with an interspike interval of <80 ms. For histological analysis of extracellular VTA neuronal recording sites, recording electrodes positions were marked with an iontophoretic deposit of pontamine sky blue dye (−20 μA, continuous current for 15 min). Brain extraction and slicing were similar to those described for cannula placement verifications. Sections were stained with neutral red and neuronal recording sites were confirmed with light microscopy. Cells recorded outside the anatomical boundaries of the VTA, as defined by Paxinos and Watson (2007), were excluded from data analysis.

### Object location memory

The object location task took place in a transparent Plexiglas arena with distinctive visual cues on each wall, with the front wall transparent. Forty rats were exposed to the arena for 20 min for two consecutive days as habituation to the context. In the training session, the subject was introduced for 5 min in the same context in the presence of a pair of identical objects. Objects were made of plastic and glass and had similar dimensions. Rats were left to explore the arena and exploration time for each of the objects was measured using a video-tracking system (ANY-maze; Stoelting). Six hours later, rats were tested for 3 min in the same context, with the same objects, but changing the position of one of them. Rats expressed memory for object recognition location, if they spent more time exploring the object in the new location, than the other one. Exploration was defined as sniffing or touching the object with the nose or forepaws. The time of exploration for each object was recorded and preference was expressed as the difference in exploration between objects according to the total exploration time.

### Social interaction and social memory testing

Social interaction and social memory procedures were performed as described previously.^19^ Testing was performed in a rectangular, three-chambered box. Forty-six rats were first placed in the middle chamber and allowed to explore for 5 min. During this session, doorways into the two side chambers were closed by plastic guillotine doors. Following habituation, an unfamiliar male rat that had no prior contact with the subject rat was placed in one of the side chambers. The location of the stranger rat in the left versus right side chamber was counterbalanced between trials. The stranger rat was enclosed in a small rectangular wire cage that allowed nose contact between the bars. Doors to the side chambers were then unblocked and the subject was allowed to explore the entire test apparatus for 8 min. An entry was defined as all four paws in one chamber. Times spent in each chamber were recorded and analyzed by a video-tracking system (ANY-maze; Stoelting). Behavioral performance was expressed using sociability scores (i.e., difference between the time spent in the stranger and empty compartments). At the end of the social interaction test, each rat was immediately tested in a subsequent 8-min session to evaluate social memory. A second, unfamiliar rat was placed in the chamber that had been empty during the first 8-min session. The tested rat had a choice between the first, already-investigated rat versus the novel unfamiliar rat. In this situation, control rats spend significantly more time with the new stranger, demonstrating a natural preference for social novelty. Measures were taken of the amount of time spent in each chamber and a social recognition score (i.e., difference between the time spent in the non-familiar rat and familiar rat chamber) was attributed to each tested rat.

### Contextual fear conditioning protocol

Sixteen rats were conditioned using a contextual fear conditioning procedure and 44 rats were conditioned using an olfactory fear conditioning as described previously.^20^ Briefly, for the context fear conditioning protocol, conditioning took place in a chamber made of transparent Plexiglas side walls with black stripes on a white background. Rats were habituated to the conditioning chamber for a 30-min session. The following day, rats were placed in the same chamber and after the first 5 min (habituation period), 10 unpredictable footshocks were delivered (1 sec, 0.8 mA). For olfactory fear conditioning, rats were habituated in a different context than the conditioning chamber for two consecutive days in 20 min sessions. The following day, rats were placed in the conditioning chamber and two odors were presented, almond and peppermint. One odor was presented with footshock (CS+; 1 sec, 0.8 mA) and the other was presented in the absence of footshock (CS−). Testing 24 h later took place in the same conditioning chamber for the context fear conditioning during which the amount of time freezing was recorded over a 10-min session; and in the habituation chamber for the olfactory fear conditioning protocol, during which CS+ was presented for a 5-min period, following presentation of CS− for another 5-min period. Amount of time freezing was recorded and computerized using a video-tracking system (ANY-maze; Stoelting). Freezing was defined as the total absence of movement (100% of the animal body) during at least 250 ms.

### Conditioned place preference

Saline or morphine injections (i.p.) were randomly paired with one of two distinct environments. Thirty-two rats received four morphine-environment and four saline-environment conditioning sessions (one session per day). We used either sub- or supra-threshold conditioning doses of morphine (0.05 vs. 5 mg/kg, i.p.), which we have demonstrated previously to produce either no morphine conditioned place preference (CPP) or significant morphine CPP, respectively.^19^ For all experiments, rats received drug or vehicle injections immediately before being placed in saline or morphine-paired environments. Intracranial microinfusions were performed before i.p. injections and given before both vehicle and morphine conditioning trials. Each conditioning session was 30 min. One week following conditioning, rats were tested (drug free) for place preference during a 10 min test and time spent in each environment were measured. CPP behavior was expressed using place preference scores (i.e., difference between times spent in morphine minus saline environments).

### Anxiety testing in the light/dark box apparatus

The light/dark box setup consisted of two compartments: one light compartment (50×25×37 cm, 250 lux) and one dark compartment (50×25×37 cm, 5 lux). The compartments were connected via a small opening allowing movement between the two boxes. Twenty-four rats were placed in the light compartment and times spent in each compartment and latency to the first entry into the light compartment were measured during a single 8-min trial using a video-tracking system (ANY-maze; Stoelting).

### Locomotor activity

Rats were microinfused with intra-vHipp PEA (1 μg/0.5 μL) 10 min before being placed in an automated open-field activity chamber (Med Associates) for a 20-min trial. Ambulatory distance was measured as an indicator of general locomotor and exploratory behavior.

### Statistical analyses

Behavioral and electrophysiological data were analyzed with a one-way analysis of variance (ANOVA), two-way ANOVA, or Student's *t*-tests where appropriate. The *post hoc* analyses were performed with Newman–Keuls tests.

## Results

### Effects of intra-vHipp GPR55 receptor activation on VTA DA neuronal activity

**FIG. 1.**
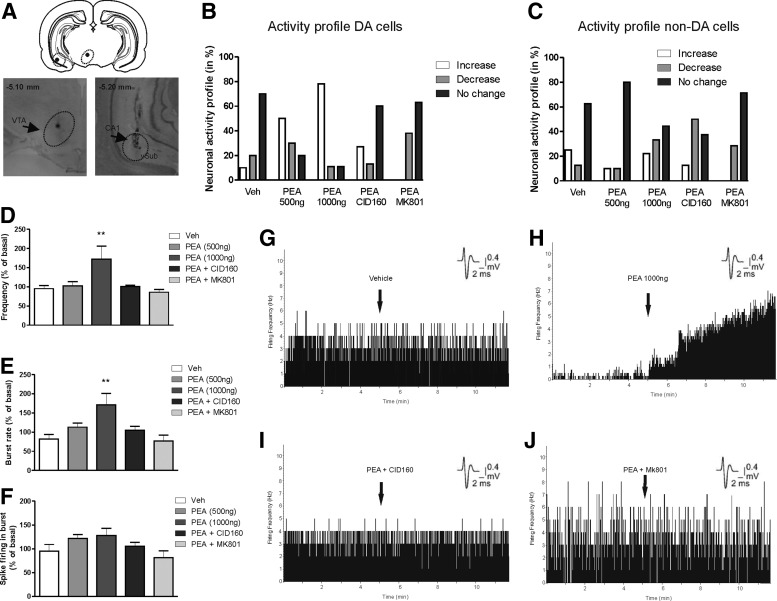
Effects of intra-vHipp PEA infusion on VTA neuronal activity. **(A)** Microphotographs and schematic reconstruction showing intra-VTA extracellular recording sites of presumptive VTA DA neurons combined with simultaneous microinfusions into the vHipp. **(B)** Population activity summary demonstrating proportions of sampled VTA DA neurons displaying relative increase, decrease, or no change in firing frequency relative to baseline. **(C)** Activity summary for sampled VTA non-DA, presumptive GABAergic neurons following intra-vHipp microinfusion treatments. Group summary showing all sampled VTA DA neurons changes from baseline following vHipp microinfusions for firing frequency **(D)**, bursting rate **(E)**, and average spikes per burst event **(F)**. Sample neuronal firing rastergrams showing representative firing rate responses following intra-vHipp vehicle **(G)**, PEA (1000 ng; 1 μg/0.5 μL) **(H)**, PEA + CID (1 μg/0.5 μL) **(I)**, or PEA + MK801 (1 μg/0.5 μL) **(J)**, microinfusions. DA, dopamine; PEA, palmitoylethanolamide; Veh, vehicle; vHipp, ventral hippocampus; VTA, ventral tegmental area.

We have previously shown that vHipp CB1R activation strongly increases VTA DA neuronal activity states.^19^ However, the potential effects of intra-vHipp GPR55 transmission on mesolimbic DA neuronal activity are currently not known. Accordingly, we examined the potential effects of intra-vHipp PEA-mediated GPR55 activation on mesolimbic DA neuron activity states (see Materials and Methods). Recording sites in the VTA and microinfusion locations in the vHipp are shown in Figure 1A. We sampled a total of *n*=50 VTA DA-like neurons [vehicle group, *n*=10 cells in 6 rats; PEA (0.5 μg/0.5 μL) group, *n*=10 cells in 5 rats; PEA (1 μg/0.5 μL) group, *n*=9 cells in 6 rats; PEA (1 μg/0.5 μL) + CID160 (1 μg/0.5 μL), *n*=13 cells in 7 rats; and PEA (1 μg/0.5 μL) + MK801 (1 μg/0.5 μL), *n*=8 cells in 5 rats].

In rats receiving intra-vHipp vehicle microinfusion, 70% of neurons did not change their firing frequency, 20% of neurons showed an increase in firing activity, and 10% demonstrated decreased activity. For rats receiving the lower PEA dose, 30% of neurons increased firing frequency, 50% of neurons showed a decrease, and 20% demonstrated no change in activity. Intra-vHipp PEA infusion of a higher dose (1 μg/0.5 μL) produce more diverse effects. Indeed, 78% of neurons increased their firing frequency, 11% decreased, and 11% demonstrated no change in activity. In contrast, only 13% of neurons increased firing frequency following microinfusion of the same high dose of PEA + coinfusion with CID160; 60% did not change their activity; and 27% decreased firing frequency. Finally, following intra-vHipp microinfusion of the higher dose of PEA + MK801, 0% of the recorded cells decreased their firing frequency, 38% of neurons increased their firing activity, and 63% demonstrated no change in activity levels. Percentages of VTA DA neurons showing increase, decrease, or no change in their activity after intra-vHipp microinfusions are summarized in Figure 1B.

Group ANOVA comparing firing frequency rates revealed a significant effect of treatment factor (*F*_4,51_=4.75, *p*=0.0026; Fig. 1D) and *post hoc* comparisons showed that rats with intra-vHipp PEA (1 μg/0.5 μL) had an average VTA DA neuronal firing frequency significantly higher than rats microinfused with vehicle (*p*=0.01), PEA (0.5 μg/0.5 μL) (*p*<0.05), PEA (1 μg/0.5 μL) + CID160 (1 μg/0.5 μL) (*p*<0.05), or PEA (1 μg/0.5 μL) + MK801 (1 μg/0.5 μL) (*p*s<0.05). Examples of typical firing frequency effects are represented in single unit histograms, after intra-vHipp microinfusions of vehicle (Fig. 1G), PEA (1 μg/0.5 μL; Fig. 1H), PEA + CID160 (Fig. 1I), and PEA + MK801 (Fig. 1J). Analysis of bursting rates revealed average changes from baseline of −3%, +42%, +120%, +5%, and +10% for rats treated with intra-vHipp vehicle, PEA (0.5 μg/0.5 μL), PEA (1 μg/0.5 μL), PEA (1 μg/0.5 μL) + CID160 (1 μg/0.5 μL), and PEA (1 μg/0.5 μL) + MK801 (1 μg/0.5 μL), respectively (Fig. 1E).

Group ANOVA comparing bursting rates before versus after infusion revealed a significant effect of treatment (*F*_4.49_=4.99, *p*=0.002), and *post hoc* comparisons showed that bursting rates were significantly increased following intra-vHipp PEA (1 μg/0.5 μL) relative to rats treated with vehicle (*p*<0.001), PEA (0.5 μg/0.5 μL) (*p*=0.017), PEA (1 μg/0.5 μL) + CID160 (1 μg/0.5 μL) (*p*=0.003), or the PEA (1 μg/0.5 μL) + MK801 (1 μg/0.5 μL) treatment (*p*s<0.001). Analysis of the number of spikes per burst revealed average changes from baseline of −5%, +22%, +61%, +5%, and 0% for rats treated with intra-vHipp vehicle, PEA (0.5 μg/0.5 μL), PEA (1 μg/0.5 μL), PEA (1 μg/0.5 μL) + CID160 (1 μg/0.5 μL), and PEA (1 μg/0.5 μL) + MK801 (1 μg/0.5 μL), respectively (Fig. 1F). ANOVA revealed no significant treatment effect on number of spikes per burst event (*F*_4,51_=2.319, *p*=0.071).

We also sampled a total of *n*=42 VTA non-DA, presumptive GABAergic neurons [see Materials and Methods; vehicle group, *n*=8 cells in 4 rats; PEA (0.5 μg/0.5 μL) group, *n*=10 cells in 6 rats; PEA (1 μg/0.5 μL) group, *n*=9 cells in 5 rats; PEA (1 μg/0.5 μL) + CID160 (1 μg/0.5 μL), *n*=8 cells in 6 rats; and PEA (1 μg/0.5 μL) + MK801 (1 μg/0.5 μL), *n*=7 cells in 3 rats]. In rats receiving intra-vHipp vehicle microinfusion, 62.5% of neurons did not change their firing frequency, 12.5% of neurons showed an increase, and 25% demonstrated decreased activity. For rats receiving the lower dose of PEA (0.5 μg/0.5 μL), 10% of the recorded cells demonstrated decreased activity, 10% showed increased firing, and 80% demonstrated no change. For rats receiving the higher dose of PEA (1 μg/0.5 μL), 22.2% of neurons decreased firing rates, 33.3% showed increased firing, and 44.4% demonstrated no change. For rats receiving the coinfusion of the PEA dose (1 μg/0.5 μL) and CID160 (1 μg/0.5 μL), 12.5% of neurons decreased their firing frequency, 50% increased their activity levels, and 37.5% showed no change in firing activity. Finally, following intra-vHipp microinfusion of the higher dose of PEA (1 μg/0.5 μL) coinfused with MK801 (1 μg/0.5 μL), 71.4% of the recorded cells did not change their firing frequency, 28.5% of neurons increased their firing activity, and 0% demonstrated increase in activity levels. Percentages of VTA non-DA neurons showing increased, decreased, or no change in activity after intra-vHipp microinfusions are represented in Figure 1C.

Taken together, these results showed that intra-vHipp GPR55 receptor activation dose-dependently increases VTA DA neuronal firing frequency and bursting activity through CB1R-independent substrate while having no significant effects on non-DA VTA neurons. Given the observed effect of vHipp GPR55R activation on DA mesolimbic neurons, we next examined the effects of intra-vHipp PEA infusions on a series of DA-mediated tasks, which have previously been reported to be modulated by cannabinoid transmission directly in the vHipp.^19,20^

### Effects of intra-vHipp GPR55 receptor activation on spatial object location memory

**FIG. 2.**
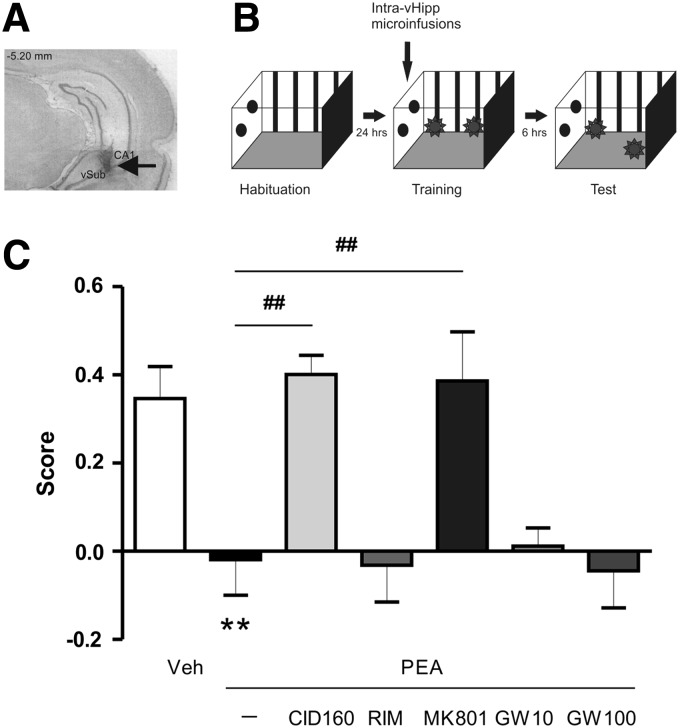
Object location memory is modulated by vHipp PEA infusion. Pretraining infusion of PEA (1 μg/0.5 μL) impairs retention of long-term object location memory, tested 6 h after training. This impairment was reversed when PEA was coinfused with GPR55 antagonist (CID160, 1 μg/0.5 μL), and NMDA antagonist (MK801, 1 μg/0.5 μL), but not by the selective PPARα antagonist, GW 6471 (10–100 ng/0.5 μL). **(A)** Photomicrograph of the vHipp showing representative microinfusion site. **(B)** Object location protocol used. **(C)** Scores of the different groups tested. *Post hoc* comparisons Newman–Keuls. **p<0.01 versus Vehicle (VEH). ^##^*p*<0.01 versus other treatments. NMDA, N-methyl-D-aspartate; PPARα, peroxisome proliferator-activated receptor alpha; RIM, Rimonabant.

We examined the potential role of GPR55R transmission in an object location recognition task (see Materials and Methods). In this task rats learn to detect the displacement of a familiar object to a novel location. This form of memory has a strong spatial memory component and requires the hippocampus.^27^ Rats were tested 6 h post-training during a 3-min test session where preferences for both objects were measured (Fig. 2B). ANOVA revealed a significant effect of treatment (F_3.421_=7.399, *p*<0.001; Fig. 2A) and *post hoc* analyses showed that intra-vHipp PEA (1 μg/0.5 μL, *p*=0.002, *n*=9) impaired the formation of object location memory, relative to vehicle controls (*n*=8). Moreover, *post hoc* analyses revealed that the effects of vHipp PEA (1 μg/0.5 μL) were reversed when it was coinfused with the selective GPR55 antagonist CID160 (1 μg/0.5 μL, *p*=0.001, *n*=8) or the NMDA antagonist MK801 (1 μg/0.5 μL, *p*=0.001, *n*=8). In contrast, no reversal effect was observed when PEA was coinfused with the CB1 antagonist RIM (0.5 μg/0.5 μL, *p*=0.915, NS, *n*=7). Since it has been shown that PEA has interactions with the nuclear receptor PPARα (24), we ran a subsequent control experiment to rule out the possibility that the effects observed following vHipp infusion of PEA may be mediated by PPARα activation, rather than GPR55R signaling. Therefore, we coinfused the effective dose of PEA (1 μg/0.5 μL) with two different doses of the selective PPARα antagonist GW 6471 (GW10, 10/0.5 μL; or GW 100, 100 ng/0.5 μL). Neither dose was observed to reverse PEA effects (GW 10; *p*=0.763, NS, *n*=7) (GW 100; *p*=0.832, NS, *n*=7). Thus, activation of vHipp GPR55Rs impairs the formation of spatial object location memory through an NMDA receptor-dependent mechanism. Given the observed lack of PPARα involvement over an order of magnitude dose in this task, no further experiments examined the effects of PPARα blockade.

### Effects of intra-vHipp GPR55 receptor activation on sociability and social recognition memory

**FIG. 3.**
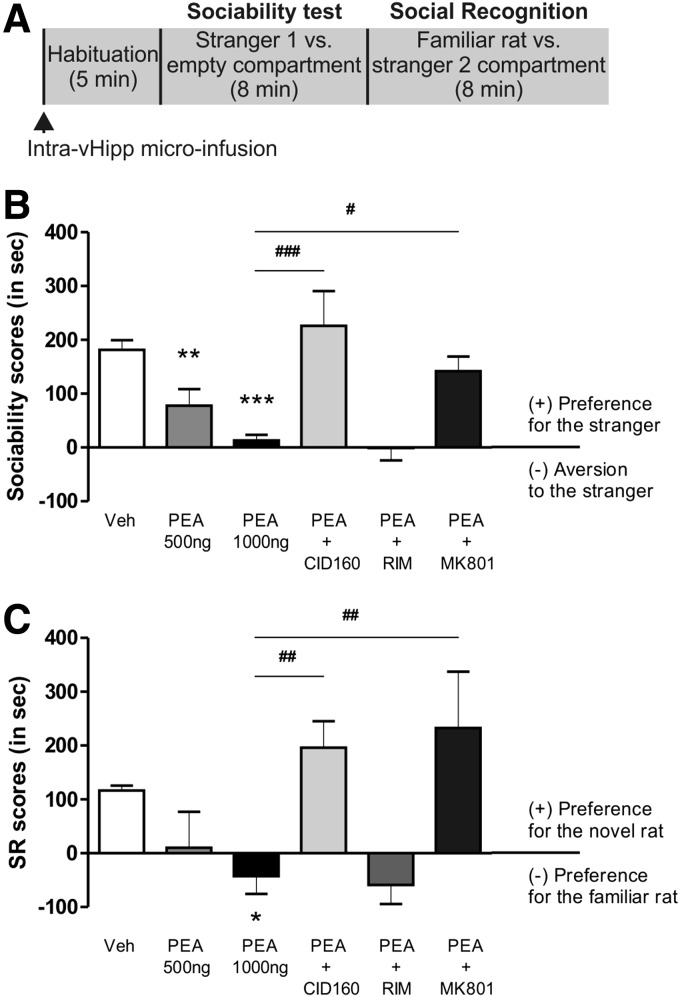
Effect of vHipp PEA infusion on social interaction/recognition tasks. **(A)** Procedural outline for social interaction and memory social recognition task. The social interaction test started with a 5 min habituation session followed by two consecutive 8 min interaction sessions (sociability and social recognition test, respectively). Intra-vHipp microinfusions were performed immediately before the habituation session. **(B)** Sociability scores. **(C)** Social recognition scores. *Post hoc* comparisons Newman–Keuls. *p<0.05, **p<0.01, ***p<0.01 versus Vehicle (VEH). ^#^*p*<0.05, ^##^*p*<0.01, ^###^*p*<0.01 versus other treatments.

We next examined the potential effects of intra-vHipp GPR55 activation on rats' natural sociability and social recognition behavior (Fig. 3A). For the social interaction test (Fig. 3B), ANOVA revealed a significant effect of treatment (F_5,45_=8.927, *p*<0.0001) on interaction times and *post hoc* analyses revealed that PEA (500 ng, 0.5 μg/0.5 μL, *p*=0.007, *n*=6), PEA (1000 ng, 1 μg/0.5 μL, *p*<0.0001, *n*=8), and PEA (1 μg/0.5 μL) + RIM (0.5 μg/0.5 μL) (*p*<0.0001, *n*=8) groups displayed sociability scores significantly lower relative to vehicle controls (*n*=8). In addition, the PEA (1 μg/0.5 μL) + MK801 (1 μg/0.5 μL) and PEA (1 μg/0.5 μL) + CID160 (1 μg/0.5 μL) groups obtained sociability scores significantly higher than rats treated with the higher dose of PEA alone (1 μg/0.5 μL) (*p*=0.006, *n*=7 and *p*<0.0001, *n*=7; respectively). For the social recognition memory phase (Fig. 3C), ANOVA revealed a significant effect of treatment (F_5,45_=4.891, *p*<0.01). *Post hoc* tests revealed that the PEA (1 μg/0.5 μL, *p*=0.044, *n*=8) and PEA (1 μg/0.5 μL) + RIM (0.5 μg/0.5 μL) (*p*=0.028, *n*=8) groups obtained social recognition score significantly lower than the vehicle group. In addition, rats microinfused with PEA (1 μg/0.5 μL) + CID160 (1 μg/0.5 μL) demonstrated social recognition scores significantly higher than rats treated with PEA (1 μg/0.5 μL, *p*=0.005) or PEA (1 μg/0.5 μL) + RIM (1 μg/0.5 μL) (*p*=0.003). Finally, the PEA (1 μg/0.5 μL) + MK801 (1 μg/0.5 μL) group demonstrated social recognition scores significantly higher than the PEA (1 μg/0.5 μL) (*p*=0.001). Thus, intra-vHipp GPR55R activation dose-dependently decreased social interaction behaviors and disrupted social recognition memory through a local NMDA receptor mechanism. Based upon this initial dose–response curve, subsequent experiments used the highest effective dose of PEA (1 μg/0.5 μL) for intra-vHipp microinfusions.

### Effects of intra-vHipp GPR55 activation on contextual versus context-independent fear memory formation

**FIG. 4.**
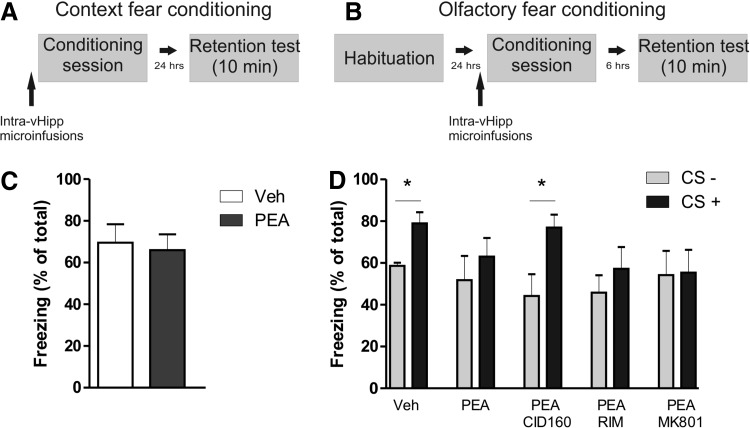
Intra-vHipp PEA infusion modulates the acquisition of olfactory fear conditioning. Rats were trained in context **(A)** or olfactory **(B)** fear conditioning with a 0.8 mA footshock. **(C)** No effect of PEA infusion was observed on the acquisition of context fear conditioning. **(D)** PEA impairs the acquisition of an olfactory fear conditioning protocol and the effect is reversed by the coinfusion of the GPR55 antagonist CID160 (1 μg/0.5 μL). *Post hoc* comparisons Newman–Keuls. *p<0.05.

We next examined the role of vHipp GPR55 receptors in modulating the formation of either contextual or context-independent olfactory fear conditioning memories (see Materials and Methods). The most effective dose of the GPR55 agonist, PEA (1 μg/0.5 μL) was microinfused into the vHipp immediately before the start of each conditioning session (Fig. 4A, B). No effect of PEA was observed when rats were tested in the contextual fear memory paradigm, as both vehicle and PEA-treated rats displayed no difference in percentages of time spent freezing during testing (t_14_=0.30, *p*=0.7663, NS, *n*=8 per group; Fig. 4C). In contrast, in separate groups tested in a context-independent fear memory paradigm, two-way ANOVA comparing percentages of time freezing revealed no significant effect of group factor (F_4.73_=0.41, *p*>0.05) and a significant effect of conditioned stimulus factor (F_1.73_=22.21, *p*<0.0001; Fig. 4D). *Post hoc* analyses revealed that Vehicle (VEH) control rats showed strong associative freezing in response to CS+ versus CS− cue presentations (*p*<0.05, *n*=7), whereas rats treated with PEA (1 μg/0.5 μL) demonstrated no associative freezing behaviors between cues (*p*>0.05, *n*=7). Coinfusion of PEA with the selective GPR55 antagonist CID160 (1 μg/0.5 μL) reversed the effects of PEA, with this group showing significant associative fear memory responses (*p*<0.05, *n*=7). However, coadministration of PEA with either RIM (0.5 μg/0.5 μL) or MK801 (1 μg/0.5 μL) failed to reverse the effects of PEA on context-independent fear memory formation (*p*s>0.05, *n*=7 per group).

### Effects of intra-vHipp GPR55 receptor activation on opiate reward memory formation

**FIG. 5.**
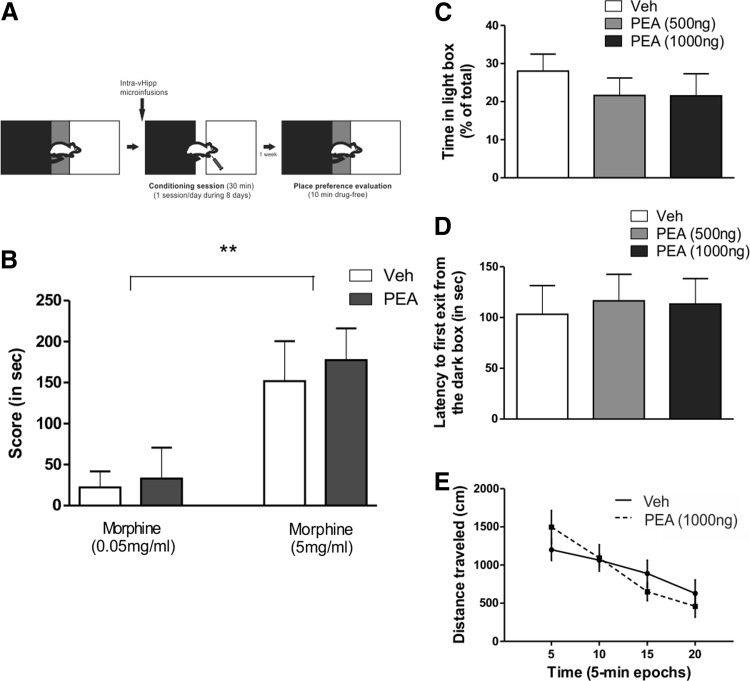
Effects of vHipp PEA infusion on morphine CPP, anxiety, and locomotion activity. **(A)** Experimental protocol. Rats were trained in a sub-threshold (morphine 0.05 mg/kg, i.p.) or supra-threshold (morphine 5 mg/kg, i.p.) morphine CPP protocol. **(B)** Vehicle or PEA (1 μg/0.5 μL) were microinfused in the vHipp immediately before each conditioning session. Memory was tested 7 days after last conditioning. There was no effect of PEA infusion in any of both conditions analyzed. *Post hoc* comparisons Newman–Keuls. **p<0.01 morphine interaction. Rats were tested on the dark/light box paradigm immediately after the infusion of vehicle, PEA 500 ng (0.5 μg/0.5 μL) or PEA 1000 ng (1 μg/0.5 μL). The time spent in the light box **(C)** and the latency for the first exit from the dark box **(D)** was measured. No significative differences were found between any of the groups tested. Rats were also tested on an open-field task and distance traveled, as a measure of locomotion activity, was measured **(E)** immediately after PEA or vehicle infusion. No effect was observed between groups. CPP, conditioned place preference.

Given previous evidence that vHipp CB1R transmission can strongly modulate opiate-related reward memory formation,^19,20^ we next investigated whether vHipp GPR55 transmission may similarly influence reward-related memory formation, using an unbiased morphine CPP procedure (see Materials and Methods). We trained separate groups of rats in a CPP protocol (Fig. 5A) using two different doses of morphine, a sub-reward threshold dose (0.05 mg/kg, i.p., *n*=8 per group) and a supra-reward threshold dose (5 mg/kg, i.p., *n*=9 per group). Rats were then tested 7 days after the last conditioning session. ANOVA comparing times spent in saline versus morphine paired environments during the CPP test phase revealed no significant treatment effect (F_1.30_=0.04, *p*=0.85, NS; Fig. 5B). Thus, intra-vHipp PEA administration had no effects on either sub or supra-threshold morphine-related associative reward memory formation.

### Effects of intra-vHipp GPR55 receptor activation on anxiety and locomotor activity

We next examined whether intra-vHipp GPR55 activation may influence anxiety levels, using the light/dark box anxiety test protocol (see Materials and Methods). ANOVA comparing percentages of time spent in the light versus dark environments (F_2,23_=0.561, *p*=0.58, NS, *n*=8 per group; Fig. 5C) or average latencies for first exits from the dark environment (F_2,23_=0.068, *p*=0.93, NS, *n*=8 per group; Fig. 5C) revealed no significant effects across any groups. Thus, intra-vHipp GPR55R activation had no apparent effects on levels of anxiety measured in the light-dark box behavioral assay. In addition, ANOVA comparing distance travelled across groups in the open field test (see Material and Methods) revealed no effect on locomotor activity (*p*>0.05; Fig. 5E) following intra-vHIPP PEA infusions (1 μg/0.5 μL).

## Discussion

The vHipp plays a vital role during the encoding of spatial memory and in the processing of affective and contextual information. Functional interactions between the vHipp, VTA, and nucleus accumbens (NAc) involving dopaminergic transmission have been shown to be critical for controlling the contextual and emotional salience of various cognitive and affective behaviors.^19,20^ Importantly, dysregulation of dopaminergic transmission within this circuitry has been proposed as a potential underlying mechanism for schizophrenia-related deficits in affective and cognitive regulation, which include social interaction deficits, distortions in affective salience processing, and memory-related impairments.^19,28^ Nevertheless, very little is known regarding the specific neuropharmacological substrates within the vHipp, which may lead to disturbances in vHipp-mesolimbic interactions.

We have demonstrated previously a critical role for cannabinoid CB1R transmission directly in the vHipp both during the regulation of mesolimbic activity patterns in the VTA>NAc pathway, and during the processing of social, opiate-related and fear-related associative learning and memory phenomena. Thus, activation of vHipp CB1R transmission was shown to potentiate the salience of normally nonsalient opiate-related reward and fear-related conditioning cues and causing significant disruptions in social interaction and recognition memory formation.^19^ These effects were dependent upon the ability of intra-vHipp CB1R stimulation to induce an overdrive of the mesolimbic DA system, indicated by increased frequency and bursting activity of VTA dopaminergic neuronal activity and increased activation of medium spiny neurons within the NAc, through dopaminergic and glutamatergic transmission substrates in the NAc.^20^ Given the relatively recent identification of the GPR55 receptor as a pharmacological substrate for the cannabinoid system,^11^ the present findings reveal for the first time the functional involvement of vHipp GPR55 transmission in the regulation of mesolimbic dopaminergic activity states. Nevertheless, future studies are required to examine the possible involvement of mesolimbic DA receptor transmission in the observed alterations in affective and cognitive processing following intra-vHIPP PEA administration.

This study employed the fatty acid amide, PEA, to characterize the local effects of GPR55 transmission in the vHipp. Although PEA interacts with other nuclear targets including PPARα nuclear,^27,28^ it displays no activity at either CB1 or CB2 receptors.^29^ This is consistent with the present findings wherein the behavioral effects of intra-vHipp PEA were selectively blocked by a GPR55, but not CB1R antagonist. Extrinsic and endogenous cannabinoids are capable of producing their effects independently of the canonical CB1/2R systems.^30^ Thus, the identification of novel, central receptor populations, which may account for the effects of either endocannabinoids or extrinsic cannabinoids such as cannabis, is important for elucidating the complexity and diversity of cannabinoid-induced neuronal and psychotropic phenomena.

The GPR55 is a GPCR found in various neural structures, including limbic areas such as the striatum, forebrain, and prefrontal cortex.^5^ While very little is known regarding the neuroanatomical distribution of GPR55 nor how GPR55 transmission may modulate specific neural circuits, recent evidence has revealed the presynaptic expression of GPR55 within the hippocampus^12^ and striatum.^11^ Furthermore, vHipp GPR55 activation has been reported to increase the probability of glutamate release by elevating presynaptic Ca2+ via activation of local Ca2+ stores in hippocampal CA3-CA1 synapses. In this study, we found that the effects of GPR55 activation were reversed by a selective GPR55 antagonist (CID160). However, local coadministration of PEA with the selective NMDA receptor antagonist, MK801, blocked the neuronal effects of vHipp GPR55 activation on VTA dopaminergic neuronal activity and rescued vHipp GPR55R-induced impairments in all tested behaviors, with the exception of context-independent fear memory formation, which appears to be mediated through a non-NMDA receptor-dependent mechanism in the vHipp. Thus, the present results are consistent with evidence that vHipp GPR55 activation increases glutamatergic release in the hippocampus, resulting in potentiated excitatory outputs from the vHipp to mesolimbic regions such as the VTA.

In terms of GPR55-mediated modulation of mesolimbic activity, while intra-vHipp CB1R activation has been reported to indirectly modulate mesolimbic DA activity by inhibiting non-DA, presumptive VTA GABAergic neuronal populations,^19^ in the present study, we found no effect of vHipp GPR55 stimulation on the spontaneous activity rates of VTA non-DA, presumptive GABAergic neurons. In contrast, GPR55 stimulation significantly increased VTA dopaminergic frequency and bursting rates through a local NMDA-receptor dependent mechanism, suggesting a separate neuronal mechanism in the vHipp for the functional effects of GPR55 versus CB1R signaling. While this study did not measure neuronal activity levels in the NAc, we have reported previously that intra-vHipp CB1R stimulation strongly increases the activity levels of medium spiny neurons (MSN) neuronal subpopulations in the shell region of the NAc through activation of excitatory glutamatergic vHipp>NAc projections. Future studies are required to examine how vHipp GPR55 stimulation may modulate intra-NAc neuronal activity states and/or whether other vHipp projection pathways (e.g., vHipp to prefrontal cortical pathways) may be modulated through GPR55 activity. In addition, given that PEA increased activity levels of dopaminergic neurons, an important question is whether blocking or inhibiting vHIPP GPR55 activity may produce opposite effects, namely, inhibiting dopaminergic neuronal activity. Again, future studies are required to address this question.

Currently, there are no published studies implicating selective disturbances in GPR55 signaling as a potential underlying variable in neuropsychiatric disorders. Nevertheless, the present and previously published reports^12^ demonstrate that hippocampal PEA infusion through GPR55 activation can potently modulate local glutamatergic signaling, and hence, associated glutamatergic projection pathways emerging from the vHipp may be relevant in this context. For example, dysregulation of glutamatergic signaling within the hippocampus is a well-established biomarker for schizophrenia-related psychosis.^31,32^ Thus, given that both CB1R and GPR55 transmission mechanisms within the vHipp appear capable of strongly modulating extrinsic dopaminergic activity states in the mesolimbic pathway, this suggests that localized cannabinoid abnormalities in the vHipp may cause dopaminergic and related affective and cognitive disturbances through either of these receptor substrates. This study adds to a growing body of evidence linking disturbances in vHipp cannabinoid receptor signaling as potential underlying mechanisms for vHipp-mesolimbic disturbances.

Interestingly, the behavioral effects of intra-vHipp GPR55 and CB1R stimulation produce both similar and divergent characteristics. For example, both vHipp CB1R and GPR55 activation induced significant disturbances in social interaction behaviors and recognition memory and blocked the formation of context-independent fear-related memories. In contrast, vHipp GPR55 activation had no impact on contextual fear memory formation, whereas vHipp CB1R activation was shown to strongly amplify the salience of contextual fear memory formation.^20^ In terms of reward-related memory processing, intra-vHipp CB1R stimulation was reported to potently increase the reward salience of morphine measured in an unbiased CPP conditioning procedure, through a DA-dependent mechanism in the NAc.^19^ In contrast, vHipp GPR55 stimulation produced no effects on morphine-reward conditioning, further demonstrating a divergent role for vHipp GPR55 versus CB1R signaling in terms of rewarding versus aversive affective processing. In addition, we observed no effects of intra-vHipp GPR55 on anxiety or locomotion levels, suggesting that vHipp GPR55R activation, in contrast to the effects of CB1R activation, do not directly influence reward or anxiety-related behavioral phenomena. Nevertheless, future studies are required to more precisely characterize the divergent effects of vHipp GPR55 versus CB1R signaling in these behavioral domains.

In summary, the present results add to a growing body of evidence demonstrating important functional roles for GPR55 signaling in cannabinoid-related neuronal and behavioral phenomena and underscore the potential for GPR55R signaling in the mediation of cannabinoid-related effects independently of the CB1/2R systems.

## Abbreviations Used

ANOVA: analysis of variance
CB1/2R: CB1 and CB2 receptor
CPP: conditioned place preference
DA: dopamine
DMSO: dimethyl sulfoxide
GPCRs: G-protein-coupled receptors
NAc: nucleus accumbens
PBS: phosphate-buffered saline
PEA: palmitoylethanolamide
PPARα: peroxisome proliferator-activated receptor alpha
RIM: Rimonabant
vHipp: ventral hippocampus
VTA: ventral tegmental area
